# Different definitions of the nonrecollection-based response option(s) change how people use the “remember” response in the remember/know paradigm

**DOI:** 10.3758/s13421-019-00938-0

**Published:** 2019-05-22

**Authors:** Helen L. Williams, D. Stephen Lindsay

**Affiliations:** 1 School of Psychology, Keele University, Dorothy Hodgkin Building, Keele, Staffordshire ST5 5BG UK; 2 University of Victoria, Victoria, British Columbia Canada

**Keywords:** Remember/know, Subjective experience, Recollection, Familiarity, Dual process

## Abstract

In the remember/know paradigm, a “know” response can be defined to participants as a high-confidence state of certainty or as a low-confidence state based on a feeling of familiarity. To examine the effects of definition on use of responses, in two experiments, definitions of “remember” and “guess” were kept constant, but definitions of “know” and/or “familiar” were systematically varied to emphasize (a) a subjective experience of high confidence without recollection, (b) a feeling of familiarity, (c) both of these subjective experiences combined within one response option, or (d) both of these experiences as separate response options. The confidence expressed in “know” and/or “familiar” definitions affected how participants used response options. Importantly, this included use of the “remember” response, which tended to be used more frequently when the nonrecollection-based middle response option emphasized a feeling of familiarity rather than an experience of “just knowing.” The influence of the definitions on response patterns was greater for items that had undergone deep rather than shallow processing, and was greater when deep-encoded and shallow-encoded items were mixed, rather than blocked, at test. Our findings fit with previous research suggesting that the mnemonic traces underlying subjective judgments are continuous and that the remember/know paradigm is not a pure measure of underlying processes. Findings also emphasize the importance of researchers publishing the exact definitions they have used to enable accurate comparisons across studies.

The remember/know (RK) paradigm has been used extensively in the past four decades to study subjective experiences of recognition (see Migo, Mayes, & Montaldi, 2012; Yonelinas, 2002, for reviews). Here, recognized items are categorized as “remember” when the participant recollects something that was thought or experienced at the time of encoding and categorized as “know” when the participant judges that the item was studied but does not recall any associated information (Tulving, 1985). How to interpret know responses has been called “the most vexatious problem in the remember/know paradigm” (Gardiner & Richardson-Klavehn, 2000, p. 238). A key contributor to this problem has been the variation in RK instructions provided to participants by different researchers, particularly with regard to how the know response option is defined.

As shown in Table 1, when instructing participants on what recognition experience should be categorized as a know response, some researchers include both familiarity and confidence in one response option, whereas others emphasize either familiarity or confidence. Arguing that confusion could arise between preexisting connotations of the confidence associated with “knowing” and how it is defined experimentally, some researchers ask participants to make remember/familiar judgments instead of remember/know, while others have used neutral terms such as Type A memory and Type B instead of remember and know (Bowler, Gardiner, & Gaigg, 2007; Bowler, Gardiner, & Grice, 2000; Levine et al., 1998; McCabe & Geraci, 2009; Wheeler & Stuss, 2003). Finally, know and familiar have also been separated as response options by some researchers (Barber, Rajaram, & Marsh, 2008; Conway, Gardiner, Perfect, Anderson, & Cohen, 1997; Dewhurst, Conway, & Brandt, 2009; Sauerland & Sporer, 2009; Wright & Sladden, 2003).

**Table 1:** Selection of quotations detailing how nonrecollective subjective experiences were described to participants in a range of studies

Authors	Response options in experiment	Representative quote and/or definitions provided to participants
Gardiner & Java (1990)	Remember Know	“Often, when *remembering* a previous event or occurrence, we consciously recollect and become aware of aspects of the previous experience. At other times, we simply *know* that something has occurred before, but without being able consciously to recollect anything about its occurrence or what we experienced at the time.” (p. 25, emphasis in original).
Rajaram (1993)	Remember Know	“‘Know’ responses should be made when you recognize that the word was in the study list but you cannot consciously recollect anything about its actual occurrence or what happened or what was experienced at the time of its occurrence. In other words, write ‘K’ (for ‘know’) when you are *certain* of recognizing the words but these words fail to evoke any specific conscious recollection from the study list.” (p. 102, emphasis added)
Bastin & Van der Linden (2003)	Remember Know Guess	“...classify a ‘yes’ response . . . as ‘Know’ if you do not remember any information associated with the face. You are *sure* that you have seen it because you have a *strong feeling of familiarity*, but you do not remember any information encoded with the face” (p. 24, emphasis added).
Bastin, Van der Linden, Michel, & Friedman (2004; recognition task)	Remember Familiar Guess	“I ask you to say ‘Familiar’ if you recognize a picture but do not remember any particular aspect of the encoding episode. But, still, you are *certain that you have seen the picture*, because you have a feeling of familiarity” (p. 168, emphasis added).
Conway, Gardiner, Perfect, Anderson & Cohen (1997)	Remember Know Familiar Guess	“You might ‘just know’ the correct answer and the alternative you have selected ‘stood out’ from the three choices available. In this case you would not recall a specific episode and instead you would simply know the answer. Answers with this basis are called KNOW answers. . . . It may be, however, that you did not remember a specific instance, nor do you know the answer. Nevertheless the alternative you have selected may seem or feel more familiar than any of the other alternatives. Answers made on this basis are called FAMILIAR answers” (p. 398).
Dewhurst & Anderson (1999)	Remember Know Guess	“A know response is one in which you recognize the item because it feels familiar in this context, but you cannot recall its actual occurrence in the earlier phase of the experiment. You recognize the item *purely on the basis of a feeling of familiarity*” (p. 667, emphasis added).
Dobbins, Kroll, & Liu (1998)	Remember Familiar	“We chose to use the word ‘familiar’ because students often confuse the more standard ‘know’ response with an expression of high confidence” (p.1309).
Donaldson, MacKenzie, & Underhill (1996)	Remember Familiar	“*...familiar* rather than *know* was used to indicate nonrecollection, because the word *know* carries a connotation of certainty that is inconsistent with a confidence rating that indicates lack of certainty. Participants find it hard to say that they are unsure that an item was there but that they know it was” (p. 487, emphasis in original).
Geraci, McCabe, & Guillory (2009)	Remember Know	Experiment 1, confidence emphasized: “You should make a know judgment if you recognize the item from the study list, but you cannot consciously recollect anything about its actual occurrence or what happened or what was experienced at the time of its occurrence. In other words, write ‘know’ when you are *certain* that you recognize the item, but it fails to evoke any specific conscious recollection from the study list” (p. 707, emphasis added). Experiment 2, confidence not emphasized: “You should respond know, by writing ‘know’ on the blank, if you think the item was studied but you cannot recollect any details about the study event” (p.708).
Harlow, MacKenzie, & Donaldson (2010)	Recollect Familiar	“Participants are trained to distinguish between familiarity and recollection (rather than the potentially misleading terms *knowing* and *remembering*)” (p. 1385, emphasis in original).
Ingram, Mickes, & Wixted (2012)	Remember Familiar	“...we exchanged *know* with *familiar* in an attempt to reduce confusion between the colloquial and experimental use of *know*” (p. 328, emphasis in original).

Adapted from Williams and Moulin (2015). Definitions from Gardiner and Java (1990) and Rajaram (1993) are provided first, as these are often referred to in the literature as “standard definitions”

To examine if differences in how remember and know responses are defined alter how participants use these response categories, Geraci, McCabe, and Guillory (2009) compared responses to two sets of RK definitions (Rajaram, 1993; Yonelinas, 2001) against responses to sure/unsure confidence judgments; the know definitions they compared are included in Table 1. They found that when confidence was emphasized in the definition of know (Experiment 1), patterns of RK judgments differed across words and nonwords, whereas confidence judgments (sure/unsure) did not (replicating the standard finding; e.g., Gardiner & Java, 1990). In contrast, when confidence was not emphasized in how know was defined, patterns were similar for remember/know and sure/unsure (Experiment 2). This demonstrates that how subjective states are defined can have important implications for interpretations of RK responses, particularly regarding the relationship between subjective experience and confidence (Geraci et al., 2009; see also McCabe & Geraci, 2009). Geraci et al.’s (2009) results are informative regarding how emphasizing confidence in the definition can alter how participants use the know response. However, because Geraci et al. compared RK instructions from different labs, the way that a remember response was defined to participants also differed across their experiments. In their Experiment 1, instructions for making a remember judgment were as follows:

You should make a remember judgment if you can consciously recollect its prior occurrence. Remember [sic] is the ability to become consciously aware again of some aspect or aspects of what happened or what was experienced at the time the word was presented (e.g., aspects of the physical appearance of the item, or of something that happened in the room, or of what you were thinking or doing at the time). In other words, the “remembered” word should bring back to mind a particular association, image, or something more personal from the time of study, or something about its appearance or position (i.e., what came before or after that word). (p. 707)

After knowing was defined, real-world remember and know-type retrieval examples were given, and the participant was asked for instances from their own life that they would classify as remember and know. In contrast, in Experiment 2, Geraci et al.’s (2009) participants received these rather more simple instructions for making remember judgments:

You should make a remember judgment if you can remember some qualitative information about the study event. This could include such things as recollecting what you were thinking about when the word was presented, what the word looked like, or what it sounded like. Moreover, you should write “remember” on the blank only if you can, if asked, tell the experimenter what you recollected about the study event. (p.708)

This instruction was followed by the know instruction (shown in Table 1) and no examples were given to, or requested from, the participant. Though both remember instructions focus on recollection of associated information from time of study, more details and examples of recollection were provided in the instructions given in their Experiment 1.

Experiments by Rotello, Macmillan, Reeder, and Wong (2005) compared traditional remember instructions (after Rajaram, 1993) against more conservative remember instructions. These specified that participants should only respond remember if they could describe specific details of the study episode and that they might need to justify their responses to the experimenter. The more conservative instructions led to fewer remember hits and fewer remember false alarms compared with traditional instructions. Thus, the patterns reported by Geraci et al. (2009) could have been influenced by the fact that remember definitions were not kept constant across their experiments.

As well as how knowing is defined, another methodological issue in the RK paradigm is whether a guess response option should be permitted (Bruno & Rutherford, 2010; Eldridge, Sarfatti, & Knowlton, 2002). A guess category was first introduced to the RK paradigm by Mäntylä (1993), but studies by Gardiner and colleagues were the first that specifically set out to compare results from RK paradigms that did versus did not allow guess responses (Gardiner, Java, & Richardson-Klavehn, 1996a; Gardiner, Kaminska, Dixon, & Java, 1996b; Gardiner, Richardson-Klavehn, & Ramponi, 1997). Gardiner, Java, et al. (1996) concluded that participants sometimes use know responses as a substitute for guesses when guessing is neither explicitly prohibited nor allowed, but when a guess response option is provided know responses demonstrate memory for the experimental episode, whereas guesses show no discriminative power. Moreover, Gardiner et al. (1997) demonstrated that effects of a response criteria manipulation on patterns of know responses by Strack and Förster (1995) were removed when a guess option was included. Strack and Förster had concluded that patterns of know responding were influenced by factors other than memory for the study episode (e.g., judgmental strategies relating to response rates). In contrast, Gardiner et al. (1997) presented evidence that only guess responses were affected by such factors.

This conclusion was supported by a qualitative analysis by Gardiner, Ramponi, and Richardson-Klavehn (1998), who found that participants’ justifications for guesses showed evidence of various inferences and other judgmental strategies that were not directly related to the individual’s memory for a studied word. For example, familiarity inferences such as “Holiday: I am eager to go on holiday, so I am not sure whether I saw it here or whether I was thinking about it”; or strategic responding, like “Harp: It seemed that there were quite a few musical instruments, so I took a guess that it came up” (both p. 8). Importantly, know justifications did not include any evidence of use of inferences or judgmental strategies. In his review, Gardiner (2008) asserted that if a researcher’s primary interest is reports of subjective experience as opposed to overall memory accuracy, then inclusion of a guess category is beneficial as it reduces the likelihood that participants will assign guesses to the know category (cf. Migo et al., 2012).

Inclusion of a guess option is also important for analysis, as it makes the RK judgment nonbinary. A guess response option was not included in the comparison of know definitions performed by Geraci et al. (2009); therefore, differences in the know definition across their experiments would not only have changed proportion of items assigned to know but they would have also necessarily changed the proportion of items assigned to remember. In the current experiments, inclusion of a guess option makes the RK judgment nonbinary, and thus if changes to the know definition alter patterns of know usage, this will not necessarily result in changes to the proportion of items assigned to remember.

The aim of the current experiments was to test how changes in how nonrecollection-based response options (know/familiar) are defined influences patterns of responses. To test this systematically, definitions of remember and guess responses were kept constant across participants, but definitions of know and/or familiar were varied to emphasize either a subjective experience of high confidence without recollection (RKG condition), a feeling of familiarity (RFG condition), both of these subjective experiences combined within one response option (RKfG condition), or both of these experiences as separate response options (RKFG condition); full definitions are shown in Table 2.

**Table 2:** Subjective experience response category definitions and the retrieval conditions in which they were used

Condition(s)	Subjective experience response category and definition
RKG RFG RKFG RKfG	REMEMBER = You have an experience of recollection for the word. This could include being consciously aware of some aspect or aspects of what was experienced at the time the word was presented in the learning phase (e.g., aspects of the physical appearance of the item, or of something that happened in the room, or of what you were thinking or doing at the time). In other words, you should choose “Remember” if you have a sense of yourself in the past and/or the word brings back to mind a particular association, image, or thought, from the time of study. *For example, if you see someone on the street, you may think, “Who is that? Oh yes, it’s the person I saw in line in the book store. I remember thinking what a funny hat they had on . . . .”*
RKG RKFG	KNOW = You feel that you just know that the word was a word you saw in the learning phase, but you cannot consciously recollect anything about its actual occurrence or what was experienced at the time of its occurrence. In other words, you should choose “Know” if you know the item was one you studied, but you cannot recollect any details associated with seeing it before. *For example, if you see someone on the street, you may think “Who is that? I know I've seen that person before, but I don't recall where that would have been…”*
RFG RKFG	FAMILIAR = You have a feeling of familiarity with the word and because of that you think that the word was one you saw in the learning phase. In other words, you should choose “Familiar” if the word feels familiar to you. *For example, if you see someone on the street you may think, “Who is that? They look very familiar . . . I don’t know why, but they seem familiar . . . .”*
RKfG	KNOW = You feel that you just know that the word was a word you saw in the learning phase, or you have a feeling of familiarity for the word, but you cannot consciously recollect anything about its actual occurrence or what was experienced at the time of its occurrence. In other words, you should choose “Know” if the word feels familiar or if you know the item was one you studied, but you cannot recollect any details associated with seeing it before. *For example, if you see someone on the street, you may think, “Who is that? I know I’ve seen that person before, but I don’t recall where that would have been . . .” or you may think “They look very familiar . . . I don’t know why, but they seem familiar . . . .”*
RKG RFG RKFG RKfG	GUESS = You do not have any memories or feelings associated with the word, and you are simply guessing that the word was one of the words you saw in the learning phase.

Our hypothesis is that if recollection is more of a continuous process (e.g., Bodner & Lindsay, 2003; Gruppuso, Lindsay, & Kelley, 1997; Gruppuso, Lindsay, & Masson, 2007; Ingram et al., 2012; Wixted & Mickes, 2010), then the extent to which the nonrecollection-based response option emphasizes confidence or familiarity could influence how participants assign items to remember. A “know” option that sounds more confident may result in fewer recognized items being assigned to “remember” than a “familiar” option that is defined in terms of a feeling of familiarity.

A second factor tested in the current experiments was whether patterns of responding differed across levels of processing. The deeper the encoding, the greater the amount of contextual elaborative information likely to be available for retrieval (via recollection) at test (e.g., Gardiner, 1988). Thus, differences in how nonrecollection-based definitions are worded may have a smaller effect on retrieval of items that were deeply encoded and therefore have more associated information available on which to make a remember response. In Experiment 1, target items either underwent shallow or deep encoding, and the recognition test included shallow-encoded targets, deep-encoded targets, and novel lure items.

## Experiment 1

### Method

#### Participants and design

Data were collected online using Qualtrics (between July 2013 and March 2016). The experiment was advertised on international psychology experiment websites, the crowdsourcing website CrowdFlower, and e-mailed to participant lists. Participants recruited from CrowdFlower were compensated $0.50 for their participation. Undergraduate students from Keele University received participation credit for their time. Data sets were excluded from analysis if proportion of false alarms (FAs) suggested participants had not understood the instructions or had been guessing (*z* scores of >±3; *n* = 9) or if they had not given appropriate justifications for their response categories at the end of the experiment (*n* = 18). This left 435 participants for analysis (302 female; mean age = 27.53 years, *SD* = 12.28, range: 18–78); a priori power to detect a small effect (Cohen’s *d* = .30) with this size sample was calculated at .99 (G*Power Version 3.1.5; Faul, Erdfelder, Lang, & Buchner, 2007). The experiment employed a mixed design with two within-subjects encoding conditions (deep and shallow processing; order counterbalanced) crossed with four between-subjects retrieval judgment conditions (RKG, RFG, RKFG, and RKfG). Participants were randomly allocated to encoding order and retrieval condition^1^; *N*s for each retrieval condition are shown in Table 3.

**Table 3:** Means [between-subjects 95% CIs] of recognition performance measures by retrieval condition, Experiment 1

Condition	*N*	Deep proportion hit	Shallow proportion hit	Proportion false alarms	*d′*	*c*
RKfG	112	.84 [.81, .87]	.56 [.52, .60]	.13 [.11, .15]	1.78 [1.66, 1.90]	0.31 [0.23, 0.39]
RKFG	105	.85 [.82, .88]	.54 [.50, .58]	.14 [.12, .16]	1.76 [1.63, 1.88]	0.33 [0.25, 0.41]
RKG	106	.85 [.82, .88]	.55 [.51, .59]	.13 [.11, .15]	1.77 [1.65, 1.90]	0.32 [0.24, 0.40]
RFG	112	.85 [.82, .88]	.56 [.52, .60]	.15 [.13, .17]	1.74 [1.61, 1.86]	0.27 [0.20, 0.35]

#### Stimuli

were medium-frequency nouns obtained from the MRC Psycholinguistic Database (mean familiarity rating of 427; range: 400–480) limited to between five and seven letters in length (mean log-HAL frequency = 7.49; English Lexicon Project). Four lists were created; each list contained 24 items (see osf.io/vecmn for stimuli lists). Each participant studied two lists, one under shallow-encoding instructions and one under deep-encoding instructions. The other two lists were used as lure stimuli on the recognition test. Use of lists as target or lure stimuli was counterbalanced across participants. Two primacy and recency fillers were shown at the start and end of each study list; half (four) of these studied fillers were included at the start of the recognition test alongside four lure fillers. Fillers were not included in analysis.

#### Procedure

After giving informed consent and providing demographic information, participants received instructions about a mental rotation task that was used as a distractor task (backwards/forwards response with letter and number stimuli rotated 0, 60, 120, 180, 240, or 300 degrees from vertical). Participants were then told that they would be shown two lists of words that they should learn for the memory test later. Participants were shown example stimuli screens for the shallow (“Does this word contain the letter *a*?” yes/no) and deep (“How pleasant is this word?” 1–6 scale) encoding conditions and then began their first study list (order of shallow and deep encoding counterbalanced across participants). For both lists, stimuli were shown individually in the center of the screen and disappeared when the participant made a response. Order of stimuli presentation was randomized anew for each participant by Qualtrics.

After studying the first list, participants were instructed about what type of judgment they would be making for the second study list (i.e., the level of encoding they had not done for the first list). At the end of the second list, participants completed the mental rotation task (12 items) as a distractor task. At the start of the test phase, participants were instructed that on the memory test half of the items would be words they had seen in the study phase—some of these would have come from the first list and some from the second list—and that the rest of the items would be new words that they had not seen in the study phase. Participants were told that each word would be shown individually on the screen and that their first task was to decide whether the word was “old” (seen in the study phase) or “new” (not seen in the study phase) and were shown an example stimulus screen (showing one of the examples they had seen prior to the study lists). Participants were instructed that if they thought a word was an “old” word, then they would be asked to make a judgment about their experience of recognizing that word. An example screen and the definitions appropriate to their retrieval condition were presented (definitions are shown in Table 2). Participants were told that the definitions would always be shown at the bottom of the page as a reminder, but that they should try to learn them so that they could make their experience judgments quickly and easily. On the recognition test, stimuli were shown individually in the center of the screen. If the participant responded that a word was “old,” the recognition experience judgment options replaced the old/new judgment options below the word with the full definitions shown at the bottom of the page. If the participant responded that a word was “new,” the next item was shown.

For all phases of the experiment, participants were instructed to respond as quickly as possible while remaining accurate. All responses were made using the mouse. In between each item, a fixation point (“+”) was shown in the center of the screen for 1 second.

To ensure that participants had been using the subjective experience response categories appropriately, at the end of the recognition test participants were asked to provide a brief description of what they meant when they used each response option; 18 participants were excluded on the basis of their justifications (see Participants section). Participants were then invited to give comments about the experiment, and the purpose of the experiment was described.

### Results and discussion

#### Recognition performance

To examine whether retrieval condition had influenced recognition performance, proportion of hits and FAs, and signal detection measures of discrimination (*d′*) and response bias (*c*), were compared across conditions; means are shown in Table 3. As calculation of *d′* and *c* involves numbers of FAs, but shallow-encoded and deeply-encoded items had not been blocked in the test phase *d′* and *c* are calculated across the whole set of studied items. The Snodgrass and Corwin (1988) correction was also employed so that *d′* and *c* could still be calculated when a participant had not made any FAs. Partial eta-squared (η_p_^2^) is reported as a measure of effect size.

A 2 (encoding condition: shallow, deep) × 4 (retrieval condition: RKfG, RKFG, RKG, RFG) mixed ANOVA on hits showed a significant main effect of encoding, with deep encoding resulting in more hits than shallow encoding, *F*(1, 431) = 968.11, *MSE* = .019, *p* < .001, η_p_^2^ = .69. There was no significant effect of retrieval condition, *F*(3, 431) = 0.81, *MSE* = .060, *p* = .97, η_p_^2^ = .004, and no significant interaction between encoding and retrieval condition, *F*(3, 431) = 0.54, *MSE* = .019, *p* =.66, η_p_^2^ = .004. One-way ANOVAs on proportion of FAs, *F*(3, 431) = .60, *MSE* = .012, *p =* .62, η_p_^2^ = .004; discrimination, *d’, F*(3, 431) = .09, *MSE* = .445, *p =* .97, η_p_^2^ = .001; and response bias, *c, F*(3, 431) = .34, *MSE* = .175, *p =* .80, η_p_^2^ = .002, also did not demonstrate a significant difference across retrieval conditions. The different subjective experience response options participants were given did not detectably alter their recognition performance

In addition to this traditional null hypothesis significance testing approach, we also computed Bayes factors to assess the strength of evidence supporting the experimental or null hypothesis for crucial analyses (see Appendix A at osf.io/vecmn/). For hits, we conducted a Bayesian ANOVA (using JASP Version 0.9; JASP Team, 2018), which compared the following four different models against a null ANOVA model: (1) a model that included only an effect of encoding, (2) a model that included only an effect of retrieval condition, (3) a model that included an effect of encoding and an effect of retrieval condition, and (4) a model that included both main effects of encoding and retrieval condition plus an effect of the interaction of those two factors. Each model has a Bayes factor associated with it, which quantifies the relative strength of evidence in support of that model in comparison to the null model (larger Bayes factors indicate stronger support for the model under consideration in comparison to the null model). We then compared these Bayes factors to see which model predicted the data the best. First, we took the model with the highest overall BF to be the best fitting model. Second, we compared how well this model fit the data in comparison with the next-best model by taking the ratio of the two model’s Bayes factors from step 1; this produces a new Bayes factor that quantifies the degree of superiority of the best model. Our analysis showed that for hits, a model that just included encoding predicted the data the best. The next-best model was the model that included both encoding and retrieval condition, but the encoding-only model was preferred over the encoding + retrieval condition model by a Bayes factor of 56.29; this gives *very strong* evidence that encoding was the only factor that influenced participants’ hit rates (classification specified by Wagenmakers et al., 2018).

For proportion of false alarms, *d′*, and *c* (and subsequent analyses), comparison of models was simpler as there was no effect of encoding to consider (i.e., the Bayesian ANOVA had to compare against the null model only a model that included an effect of retrieval condition). The model that included the retrieval condition was preferred over the null model by a Bayes factor of: 0.021 for false alarms, 0.015 for response bias (*c*), and 0.010 for discrimination (*d′*), giving very strong evidence in support of the null hypothesis that retrieval condition had no effect on these measures.

#### Use of subjective experience response options

**Fig. 1:**
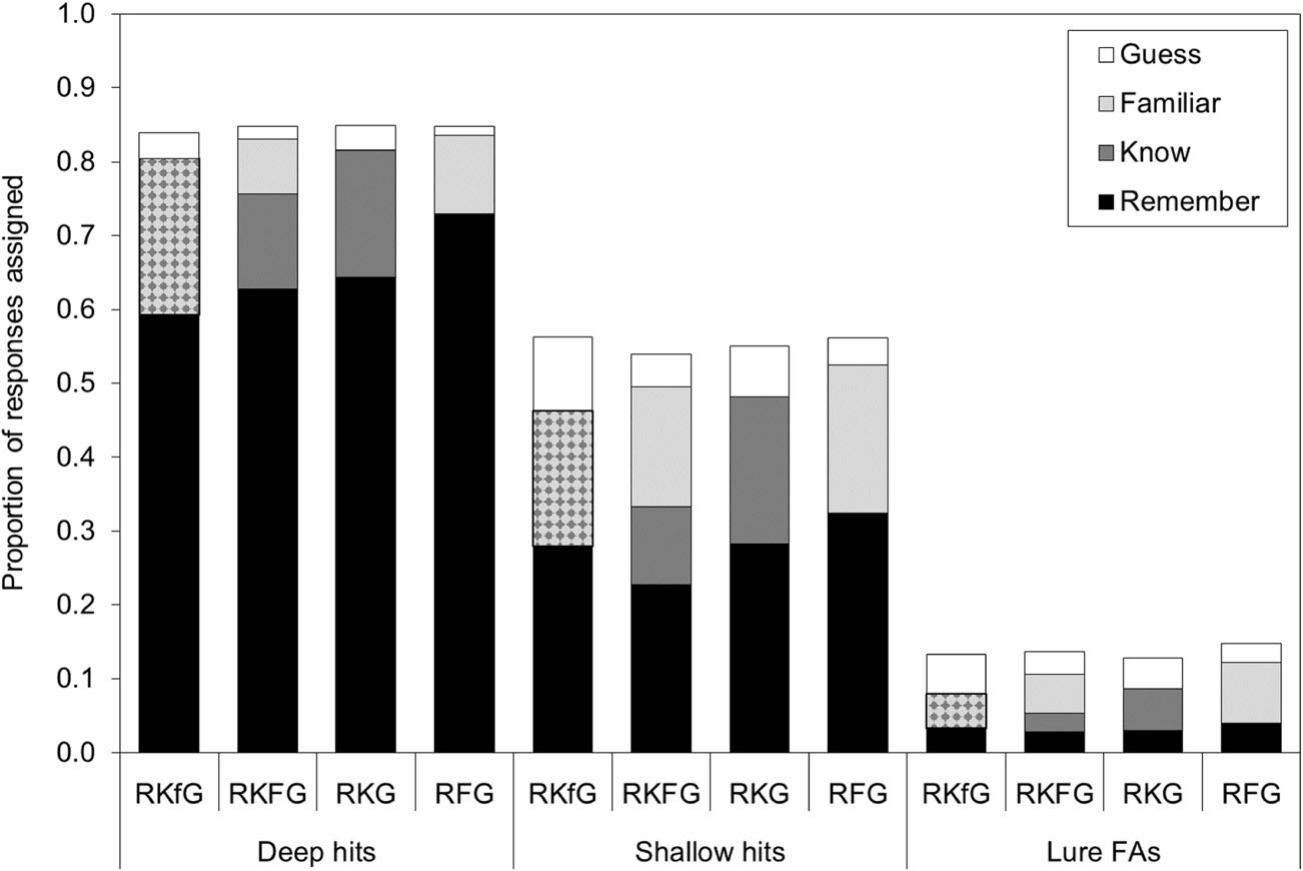
Mean proportions of hits and false alarms (FAs) assigned to Remember, Know, Familiar, and Guess in each of the retrieval conditions in Experiment 1. A table of means with their 95% CIs is available in the supplemental files (osf.io/vecmn)

To directly compare the proportions of responses assigned to the nonrecollection-based (“middle”) response option(s), proportions assigned to know and/or familiar responses were allocated to a new “KF” variable in the following ways for the different retrieval conditions: RKFG = proportion K + proportion F summed; RKG = proportion K; RFG = proportion F; RKfG = proportion Kf. To examine whether the different response option definitions changed participants’ responding, separate 4 (retrieval condition: RKfG, RKFG, RKG, RFG) one-way ANOVAs were conducted on the proportion of items assigned to remember, the pooled KF category, and guess for correctly recognized items studied under shallow or deep encoding conditions, and for FAs on lure items. Data are shown in Fig. 1 (including proportions assigned to raw know and/or familiar response options).

#### Deep encoding

For items that had undergone deep encoding, there was a significant main effect of retrieval condition on proportion assigned to remember, *F*(3, 431) = 5.12, *MSE* = .074, *p* = .002, η_p_^2^ = .034. Pairwise comparisons using a Bonferroni-adjusted alpha level of .008 for six comparisons indicated that the proportion of hits assigned to remember was significantly larger in the RFG condition than in the RKfG condition (*p* < .001), or in the RKFG condition (*p* = .006); the proportion of hits assigned to remember was numerically higher in the RFG condition than in the RKG condition, but that difference was not significant (*p* = .021); no other comparisons were significant (smallest *p* = .17). Bayesian ANOVA resulted in a Bayes factor of 8.82 for a model including retrieval condition, providing moderate evidence that retrieval condition influenced proportion of deep hits assigned to remember.

For the pooled KF response category, there was a significant main effect of retrieval condition on proportion assigned to KF, *F*(3, 431) = 6.63, *MSE* = .039, *p* < .001, η_p_^2^ = .044. A larger proportion of hits were assigned to KF in the RKfG condition (*M* = .21, 95% CI [.18, .25]) or the RKFG condition (*M* = .20, 95% CI [.17, .24]) than in the RFG condition (*M* = .11, 95% CI [.07, .14]; both *p*s < .001). The proportion of hits assigned to KF was also numerically higher in the RKG condition (*M* = .17, 95% CI [.13, .21]) than in the RFG condition, but using the Bonferroni-adjusted alpha level, the difference was not significant (*p* = .013). The Bayes factor was 66.52 for a model including retrieval condition, providing very strong evidence that retrieval condition influenced proportion of deep hits assigned to KF.

There was a significant main effect of retrieval condition on proportion assigned to guess, *F*(3, 431) = 5.71, *MSE* = .002, *p =* .001, η_p_^2^ = .038. A greater proportion of items were assigned to guess in the RKfG condition and the RKG condition compared to in the RFG condition (*p* ≤ .001 and *p* = .002, respectively). The proportion of hits assigned to guess was numerically higher in the RKfG condition and the RKG condition than in the RKFG condition, but using the Bonferroni-adjusted alpha level, these differences were not significant (*p* = .010 and *p* = .014, respectively); no other comparisons were significant (smallest *p* = .52). Fewer than .04 of responses were assigned to guess in any of the retrieval conditions. The Bayes factor was 19.17 for a model including retrieval condition, providing strong evidence that retrieval condition influenced proportion of deep hits assigned to guess.

#### Shallow encoding

For items that had undergone shallow encoding, there was a significant main effect of retrieval condition on proportion assigned to remember, *F*(3, 431) = 3.73, *MSE* = .046, *p* = .011, η_p_^2^ = .025. A larger proportion of hits were assigned to remember in the RFG condition than in the RKFG condition (*p* = .001); no other comparisons were significant (smallest *p* = .06) for the RKG versus RKFG comparison. The Bayes factor was 1.36 for a model including retrieval condition, providing only anecdotal evidence that retrieval condition influenced proportion of shallow hits assigned to remember.

For the pooled KF response category, there was a significant main effect of retrieval condition on proportion assigned to KF, *F*(3, 431) = 7.10, *MSE* = .022, *p* < .001, η_p_^2^ = .047. A larger proportion of hits were assigned to KF in the RKFG condition (*M* = .27, 95% CI [.24, .30]) than in the RKfG condition (*M* = .18, 95% CI [.16, .21]; *p* < .001), RKG condition (*M* = .20, 95% CI [.17, .23]; *p* = .001), or the RFG condition (*M* = .20, 95% CI [.17, .23]; *p* = .001). The Bayes factor was 120.28 for a model including retrieval condition, providing extremely strong evidence that retrieval condition influenced proportion of shallow hits assigned to KF.

There was a significant main effect of retrieval condition on proportion assigned to guess, *F*(3, 431) = 14.12, *MSE* = .006, *p* < .001, η_p_^2^ = .089. A greater proportion of items were assigned to guess in the RKfG condition compared with any other condition (RKfG vs. RKFG: *p* < .001; RKfG vs. RKG: *p* = .006; RKfG vs. RFG: *p* < .001). Also, a greater proportion of items were assigned to guess in the RKG condition compared with the RFG condition (*p* = .002) and the RKFG condition (*p* = .022), though this latter comparison did not meet the Bonferroni-adjusted alpha level. More items were assigned to guess that had undergone shallow encoding than had undergone deep encoding, but still fewer than .10 of responses were assigned to guess in any retrieval condition. The Bayes factor was 1.218 × 10^6^ for a model including retrieval condition, providing extremely strong evidence that retrieval condition influenced proportion of shallow hits assigned to guess.

#### False alarms to lures

For lure items, the main effect of retrieval condition on proportion of FAs assigned to remember was not significant, *F*(3, 431) = 0.89, *MSE* = .003, *p* = .45, η_p_^2^ = .006. The Bayes factor was 0.03 for a model including retrieval condition, providing strong evidence that retrieval condition did not influence proportion of false alarms assigned to remember.

For the pooled KF response category, there was a significant main effect of retrieval condition on proportion FAs assigned to KF, *F*(3,431) = 6.67, *MSE* = .005, *p* = .001, η_p_^2^ = .044. A larger proportion of FAs were assigned to KF in the RKFG condition (*M* = .08, 95% CI [.06, .09]) and the RFG condition (*M* = .08, 95% CI [.07, .10]) than in the RKfG condition (*M* = .05, 95% CI [.03, .06]; *p* = .001 and *p* < .001, respectively). The proportion of FAs assigned to KF was also higher in the RFG condition than in the RKG condition (*M* = .06, 95% CI [.04, .07]; *p* = .006). The proportion of FAs assigned to KF was also numerically higher in the RKFG condition than in the RKG condition, but using the Bonferroni-adjusted alpha level this difference was not significant (*p* = .028). The Bayes factor was 69.37 for a model including retrieval condition, providing very strong evidence that retrieval condition influenced proportion of false alarms assigned to KF.

There was a significant main effect of retrieval condition on proportion assigned to guess, *F*(3, 431) = 7.13, *MSE* = .002, *p* < .001, η_p_^2^ = .047. A greater proportion of FAs were assigned to guess in the RKfG condition compared with in the RFG condition or the RKFG condition (both *p*s < .001). The proportion of FAs assigned to guess was numerically higher in the RKG condition than in the RFG condition, but using the Bonferroni-adjusted alpha level this difference was not significant (*p* = .013); no other comparisons were significant (smallest *p* = .061 for RKG vs. RKFG). The Bayes factor was 127.40 for a model including retrieval condition, providing extremely strong evidence that retrieval condition influenced proportion of false alarms assigned to guess.

Overall, different definitions for the nonrecollection-based “middle” response option(s) resulted in a significant main effect of retrieval condition for all DVs: proportion of items assigned to remember, KF, and guess (except for FAs on lure items for which there was no main effect on remember responses). After deep encoding, more remember responses were made when the “middle” response definition was based on a feeling of familiarity only, rather than including a sense of “just knowing” or high confidence without recollection. This increase in remember responses was accompanied by a decrease in responses based on familiarity (F response in RFG condition). In addition, more guess responses were made when only one “middle” response option was provided and the definitions of this option included high confidence without recollection, though guess responses were very infrequent overall.

For shallow-encoded items, the increase in remember responses was only evident when comparing RFG and RKFG conditions, suggesting that when encoding was less elaborate and less associated information was available for retrieval, participants use the separate know and familiar options (in the RKFG condition) more. Guess responses were again infrequent, but for shallow-encoded items, more guess responses were made when the “middle” response option definition included high confidence without recollection. These patterns for KF and guess responses for shallow-encoded items were then repeated for FAs to lure items. In sum, the overall influence of the different definitions on response patterns appears to be stronger after deep encoding compared with shallow encoding.

## Experiment 2

In Experiment 1, target items either underwent shallow or deep encoding, and the recognition test included shallow-encoded targets, deep-encoded targets, and novel lure items, so recognition of items that had been shallowly or deeply encoded took place in the context of the other type of item. However, previous research has shown that test-list context affects how participants use response options in an RK task (Bodner & Lindsay, 2003; Tousignant & Bodner, 2012; Tousignant, Bodner, & Arnold, 2015). Thus, in Experiment 2, we used a blocked procedure in which the shallow and deep encoding phases were each followed by their own separate recognition test. If test-list context influences how participants define remembering and knowing for themselves during the task (Bodner & Lindsay, 2003), then removing the context of the “other” type of encoded item in our recognition test may lead to different patterns of responding across the different retrieval judgment conditions (RKG, RFG, RKfG, RKFG) on the deep and shallow tests.

### Method

Details of participant recruitment, experimental design, stimuli, and procedure were identical to Experiment 1, except where noted.

#### Participants

Data were collected online using Qualtrics (between February 2015 and April 2017). The experiment was advertised on international psychology experiment websites, and undergraduate students from Keele University received participation credit for taking part. Data sets were excluded from analysis if proportion of FAs suggested participants had not understood the instructions or had been guessing (*z* scores of >±3 for FAs in either block of the experiment, *n* = 13). This left 431 full data sets for analysis (328 female; mean age = 21.50 years, *SD* = 7.72, range: 18–69).

#### Procedure

The procedure was identical to that of Experiment 1, except that the shallow and deep encoding conditions each had their own separate recognition test (i.e., participants completed two study-test blocks: one shallow-processing block and one deep-processing block, order of blocks counterbalanced across participants). The distractor task was also changed from a mental rotation task to mathematical sums; participants completed a set of distractor sums between each study and test phase and also between the test phase for Block 1 and the study phase for Block 2. After the final recognition test, participants were invited to give comments about the experiment and the purpose of the experiment was described. To minimize completion time in this more complex two-block procedure, justifications of response categories were not requested in this experiment.

### Results and discussion

#### Recognition performance

Recognition performance measures were again compared across retrieval conditions; means are shown in Table 4. Although proportion hits and FAs for deeply encoded items are similar in this experiment compared with Experiment 1, the separation of the shallow and deep tasks into two separate study-test blocks apparently improved performance on the shallow recognition test as proportion hit increased by ~.14 (compare Tables 3 and 4).

**Table 4:** Means [between-subjects 95% CIs] of recognition performance measures by retrieval condition, Experiment 2

Condition	*N*	Deep encoding				Shallow Encoding			
		Prop. hit	Prop. FA	*d′*	*c*	Prop. hit	Prop. FA	*d′*	*c*
RKfG	111	.88 [.84, .91]	.08 [.06, .09]	2.82 [2.65, 3.00]	.08 [−.01, .16]	.72 [.68, .76]	.17 [.15, .20]	1.64 [1.48, 1.80]	.20 [.11, .29]
RKFG	108	.86 [.82, .89]	.06 [.04, .08]	2.81 [2.64, 2.99]	.17 [.08, .25]	.68 [.64, .72]	.14 [.11, .16]	1.71 [1.55, 1.87]	.33 [.24, .43]
RKG	103	.90 [.86, .94]	.07 [.06, .09]	2.91 [2.72, 3.09]	.06 [−.03, .15]	.69 [.65, .74]	.15 [.12, .17]	1.67 [1.51, 1.83]	.29 [.19, .37]
RFG	109	.86 [.85, .92]	.07 [.05, .09]	2.88 [2.70, 3.06]	.08 [−.01, .17]	.68 [.64, .72]	.15 [.13, .18]	1.62 [1.46, 1.78]	.30 [.21, .39]

Separate 2 (encoding condition: shallow, deep) × 4 (retrieval condition: RKfG, RKFG, RKG, RFG) mixed ANOVAs were conducted on proportion of hits and FAs, and measures *d'* and *c*. Each ANOVA showed a significant main effect of encoding, as deep encoding resulted in more hits, *F*(1, 427) = 407.06, *MSE* = 0.019, *p* < .001, η_p_^2^ = .49; fewer FAs, *F*(1, 427) = 193.69, *MSE* = .008, *p* < .001, η_p_^2^ = .31; better discrimination, *d'*, *F*(1, 427) = 721.54, *MSE* = 0.426, *p* < .001, η_p_^2^ = .63; and a less conservative response bias, *c*, *F*(1, 427) = 75.89, *MSE* = 0.096, *p* < .001, η_p_^2^ = .15, than shallow encoding. No significant main effect of retrieval condition was observed for any measure: proportion hit, *F*(3, 427) = 0.62, *MSE* = 0.067, *p* = .61, η_p_^2^ = .004; proportion FAs, *F*(3, 427) = 1.38, *MSE* = 0.017, *p* = .25, η_p_^2^ = .010; discrimination, *d'*; *F*(3, 427) = 0.11, *MSE* = 1.15, *p* = .95, η_p_^2^ = .001; response bias, *c*, *F*(3, 427) = 1.44, *MSE* = 0.33, *p* = .23, η_p_^2^ = .010; and there was no interaction between encoding condition and retrieval condition on any measure: proportion hit, *F*(3, 427) = 1.49, *MSE* = 0.019, *p* =.22, η_p_^2^ = .010; proportion FAs, *F*(3, 427) = 0.71, *MSE* = 0.008, *p* = .55, η_p_^2^ = .005; discrimination, *d', F*(3, 427) = 0.60, *MSE* = 0.43, *p* = .62, η_p_^2^ = .004; response bias, *c*, *F*(3, 427) = 1.21, *MSE* = 0.096, *p* = .31, η_p_^2^ = .008. In this experiment the different subjective experience response options participants were given again did not affect their recognition performance, discrimination ability, or response bias.

We again computed Bayes factors for these analyses (see Appendix B at osf.io/vecmn). For proportion hit, proportion FAs, *d'*, and *c*, each analysis confirmed that a model that included only encoding predicted the data the best. For each measure, the next best model was the model that included both the encoding and retrieval condition, but the encoding-only model was preferred over the encoding + retrieval condition model by a Bayes factor of 9.51 for response bias (*c*), 15.60 for proportion FAs, 23.24 for proportion hit, and 65.91 for discrimination (*d'*); this gives strong to very strong evidence that encoding was the only factor that influenced performance on each recognition measure.

#### Use of subjective experience response options

**Fig. 2:**
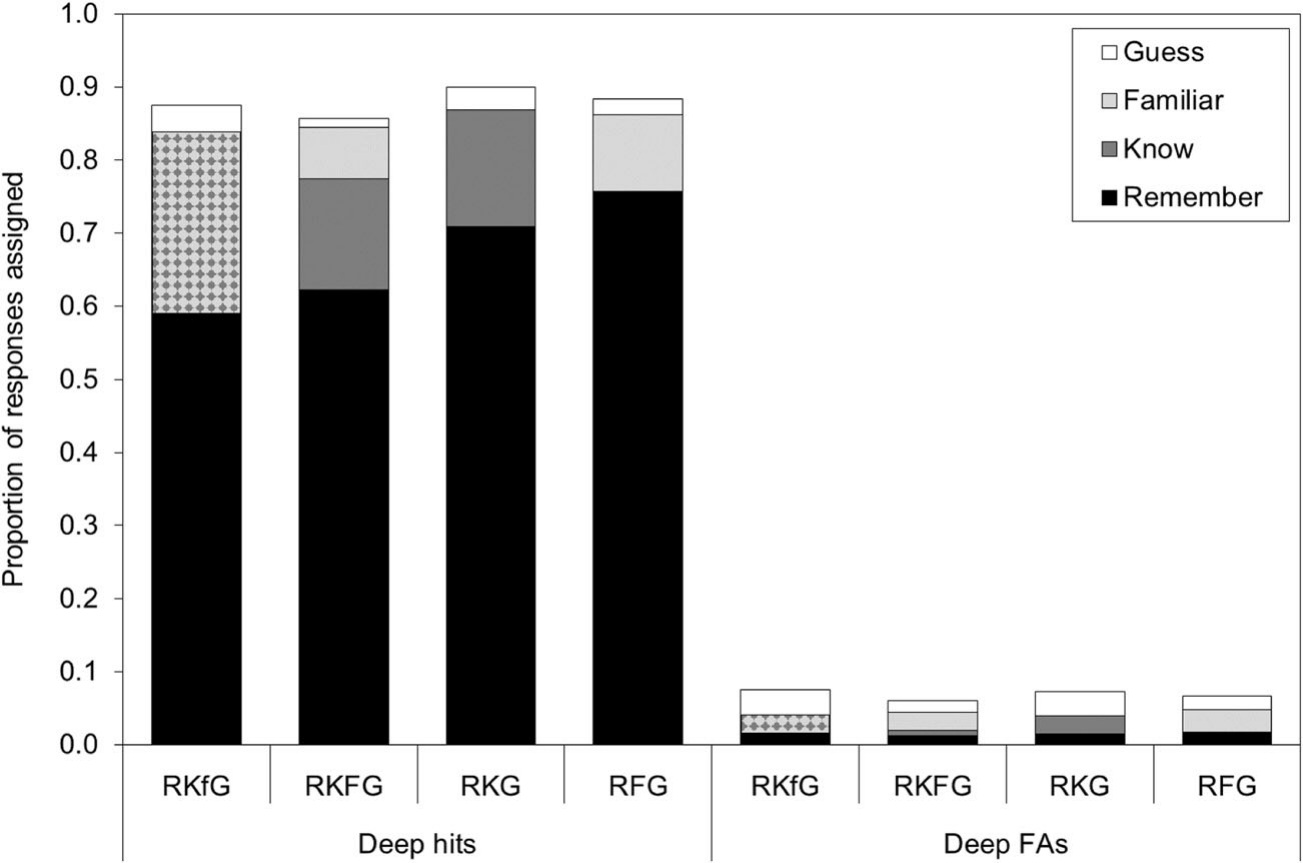
Mean proportions of hits and FAs on the deep recognition test assigned to remember, know, familiar, and guess in each of the retrieval conditions in Experiment 2. A table of means with their 95% CIs are available in supplemental files at osf.io/vecmn

**Fig. 3:**
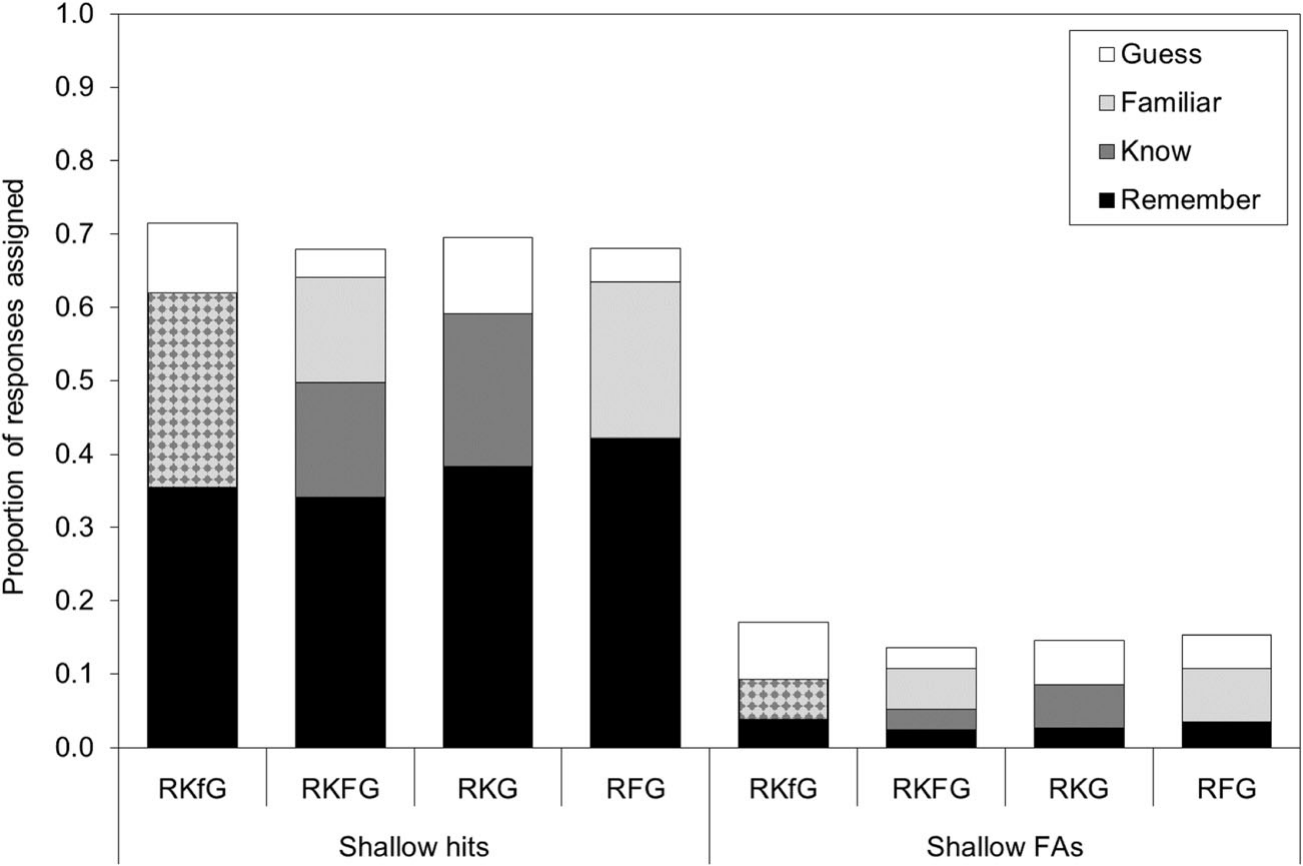
Mean proportions of hits and FAs on the shallow recognition test assigned to remember, know, familiar, and guess in each of the retrieval conditions in Experiment 2. A table of means with their 95% CIs are available in supplemental files at osf.io/vecmn

To examine whether the different response option definitions changed participants’ responding, separate 4 (retrieval condition: RKfG, RKFG, RKG, RFG) one-way ANOVAs were conducted on the proportion of items assigned to remember, the pooled KF response option, and guess, this time for both correctly recognized items and FAs on lures in the shallow and deep recognition tests; data for deep encoding are shown in Fig. 2 and data for shallow encoding are shown in Fig. 3.

#### Deep encoding—hits

For items that had undergone deep encoding, there was a significant main effect of retrieval condition on proportion assigned to remember, *F*(3, 427) = 7.19, *MSE* = .091, *p* < .001, η_p_^2^ = .048. Pairwise comparisons using a Bonferroni-adjusted alpha level of .008 for six comparisons indicated that a larger proportion of hits were assigned to remember in the RFG condition than in the RKfG condition (*p* < .001), or in the RKFG condition (*p* = .001). The proportion of hits assigned to remember was also higher in the RKG condition than in the RKfG condition (*p* =.004), and the RKFG condition (*p* = .035), but the latter difference was not significant. No other comparisons were significant (smallest *p* = .25). The Bayes factor was 137.83 for a model including retrieval condition, providing extremely strong evidence that retrieval condition influenced proportion of deep hits assigned to remember.

For the pooled KF response category there was a significant main effect of retrieval condition on proportion assigned to KF, *F*(3, 427) = 9.54, *MSE* = .048, *p* < .001, η_p_^2^ = .063. A larger proportion of hits were assigned to KF in the RKfG condition (*M* = .25, 95% CI [.21, .29]) than in the RFG condition (*M* = .11, 95% CI [.06, .15]; *p* < .001) or the RKG condition (*M* = .16, 95% CI [.12, .20]; *p* = .003). Similarly, the proportion of hits assigned to KF was higher in the RKFG condition (*M* = .22, 95% CI [.18, .27]) than in the RFG condition (*p* < .001), and the RKG condition (*p* = .035), but using the Bonferroni-adjusted alpha level the latter difference was not significant. No other comparisons were significant (smallest *p* = .08) for the RFG versus RKG comparison. The Bayes factor was 3,090.75 for a model including retrieval condition, providing extremely strong evidence that retrieval condition influenced proportion of deep hits assigned to KF.

There was also a significant main effect of retrieval condition on proportion assigned to guess, *F*(3, 431) = 4.20, *MSE* = .003, *p* = .006, η_p_^2^ = .029. A smaller proportion of hits were assigned to guess in the RKFG condition compared with in the RKfG condition (*p* = .001), or the RKG condition (*p* = .009). Overall, fewer than .04 of responses were assigned to guess in any retrieval condition. The Bayes factor was 2.60 for a model including retrieval condition, providing only anecdotal evidence that retrieval condition influenced proportion of deep hits assigned to guess.

#### Deep encoding—false alarms

For lures on the deep recognition test, the main effect of retrieval condition was not significant for proportion assigned to remember, *F*(3, 431) = 0.31, *MSE* = .001, *p* = .82, η_p_^2^ = .002, or to the pooled KF response category, *F*(3, 431) = 0.59, *MSE* = .002, *p =* .62, η_p_^2^ = .004. Fewer than .02 FAs were assigned to remember and fewer than .04 to KF on this test. For remember the Bayes factor was 0.014 for a model including retrieval condition, and for KF the Bayes factor was 0.021 for a model including retrieval condition, providing very strong evidence that retrieval condition did not influence proportion of deep FAs assigned to remember or KF.

Retrieval condition did produce a significant main effect on proportion of FAs assigned to guess, *F*(3, 431) = 4.39, *MSE* = .002, *p* = .005, η_p_^2^ = .030. A greater proportion of FAs were assigned to guess in the RKfG condition and the RKG condition compared with in the RKFG condition (*p* = .004 and *p* = .008, respectively); the proportions assigned to guess in the RKfG and RKG conditions were also numerically higher than the proportion assigned to guess in the RFG condition, but using the Bonferroni-adjusted alpha level these differences were not significant (*p* = .016 and *p* = .03, respectively). Overall, fewer than .04 of responses were assigned to guess in any retrieval conditions. The Bayes factor was 3.34 for a model including retrieval condition, providing (barely) moderate evidence that retrieval condition influenced proportion of deep FAs assigned to guess.

In this experiment, in which deep and shallow items underwent separate recognition tests, the different definitions for the nonrecollection-based “middle” response option(s) again resulted in a significant main effect of retrieval condition for deeply encoded hits assigned to remember, KF, and guess. More remember responses were made when only one “middle” response option was given (RFG and RKG) compared with when two options were provided (RKFG) or when the middle option included both knowing and familiarity (RKfG). The concomitant changes in proportion assigned to KF occurred, but also fewer guess responses were made when four response options were provided (RKFG) and the “other” option did not include high confidence without recollection. FAs were rare in the deep condition, and the different definitions only resulted in changes to proportions of guess responses, with fewer FAs assigned to guess in the RKFG condition where two middle response options could be used—know and familiar.

#### Shallow encoding—hits

For items that had undergone shallow encoding, overall levels of remember judgments for shallow hits were ~.10 higher in the current experiment where the recognition tests for deep and shallow were conducted separately compared, with in Experiment 1 where shallow and deep items were on the same recognition test (compare Figs. 1 and 2). In this experiment, the main effect of retrieval condition on proportion assigned to remember was not significant, *F*(3, 427) = 2.32, *MSE* = .058, *p* = .075, η_p_^2^ = .016. Here, different definitions of know and/or familiar did not significantly affect use of the remember response for correctly recognized shallow items. The Bayes factor was 0.21 for a model including retrieval condition, providing moderate evidence in support of the null hypothesis that retrieval condition did not influence proportion of shallow hits assigned to remember.

For the pooled KF response category, there was a significant main effect of retrieval condition on proportion assigned to KF, *F*(3, 427) = 7.86, *MSE* = .026, *p* < .001, η_p_^2^ = .052. A larger proportion of hits were assigned to KF in the RKFG condition (*M* = .30, 95% CI [.27, .33]) than in the RKG condition (*M* = .21, 95% CI [.18, .24]) or the RFG condition (*M* = .21, 95% CI [.18, .24]; both *p*s < .001). The proportion of hits assigned to KF in the RKfG condition was also numerically higher than the proportion assigned to RFG and RKG, but using the Bonferroni-adjusted alpha level these differences were not significant (*p* = .016 and *p* = .009, respectively). The Bayes factor was 331.62 for a model including retrieval condition, providing extremely strong evidence that retrieval condition influenced proportion of shallow hits assigned to KF.

There was a significant main effect of retrieval condition on proportion assigned to guess, *F*(3, 427) = 17.03, *MSE* = .007, *p* < .001, η_p_^2^ = .107. A greater proportion of hits were assigned to guess in the RKfG condition and the RKG condition compared with in the RFG and RKFG conditions (all *p*s ≤ .001). The Bayes factor was 4.915 × 10^7^ for a model including retrieval condition, providing extremely strong evidence that retrieval condition influenced proportion of shallow hits assigned to guess.

#### Shallow encoding—false alarms

For lures on the shallow recognition test, the main effect of retrieval condition on proportion assigned to remember was not significant, *F*(3, 427) = 1.10, *MSE* = .004, *p* = .35, η_p_^2^ = .028. The Bayes factor was 0.041 for a model including retrieval condition, providing strong evidence that retrieval condition did not influence proportion of shallow FAs assigned to remember.

The main effect of retrieval condition was significant for shallow FAs assigned to the pooled KF category, *F*(3, 427) = 3.22, *MSE* = .006, *p* = .023, η_p_^2^ = .022. A greater proportion of FAs were assigned to KF in the RKFG condition (*M* = .08, 95% CI [.07, .10]) than in the RKfG condition (*M* = .06, 95% CI [.04, .07]; *p* = .006), or the RKG condition (*M* = .06, 95% CI [.04, .07]; *p* = .015), though this latter difference did not reach the Bonferroni-adjusted significance level. Moreover, the Bayes factor was 0.703 for a model including retrieval condition, providing only anecdotal evidence that retrieval condition did not influence proportion of shallow FAs assigned to KF.

There was a significant main effect of retrieval condition on proportion of FAs assigned to guess, *F*(3, 427) = 11.33, *MSE* = .004, *p* < .001, η_p_^2^ = .074. A greater proportion of FAs were assigned to guess in the RKfG condition than in the RFG or RKFG conditions, and were assigned to guess in the RKG condition compared with in the RKFG condition (all *p*s < .001). The Bayes factor was 32,198.99 for a model including retrieval condition, providing extremely strong evidence that retrieval condition influenced proportion of shallow FAs assigned to guess.

On the shallow recognition test, the different definitions for the nonrecollection-based middle response option(s) did not alter the proportion of responses assigned to remember, either for hits or FAs, but did alter use of the KF and guess response options. For both hits and FAs, more KF responses and fewer guess responses were made when two middle response options were provided (RKFG) or when the middle option consisted solely of familiarity (RFG). When the middle option definition was based on high confidence without recollection, more guesses were made.

## General discussion

Patterns of subjective experience judgments were compared in two experiments in which shallowly and deeply encoded items were either tested in one recognition test (Experiment 1) or in separate blocked recognition tests (Experiment 2). In both experiments, participants made their judgments using one of four variants of RK response options; how remember and guess were defined was kept consistent across those four conditions, but definitions of the other response option(s) were varied systematically. As a brief reminder, definitions of know and/or familiar emphasized either a subjective experience of high confidence without recollection (RKG condition), a feeling of familiarity (RFG condition), both of these experiences within one response option (RKfG condition), or both of these experiences as separate response options (RKFG condition).

The different retrieval options did not affect overall recognition performance. But how the nonrecollective “middle” option(s) were defined did influence how participants used the response categories, including the remember category. That pattern was more evident when shallow-encoded and deep-encoded items were both presented (alongside lure items) within the same recognition test (Experiment 1). When the shallow and deep tests were blocked (Experiment 2), the different definitions altered response patterns to a smaller extent.

That the different retrieval conditions did not influence overall recognition was expected; we did not predict that altering how postrecognition subjective response options were defined would change participant’s ability to discriminate old from new items. In the study of subjective experiences, researchers typically assume that what is actually experienced at recognition is the same across standard (old/new) recognition tasks and tasks that require a postrecognition assessment of the subjective nature of the retrieval experience (e.g., RK tasks). However, a few researchers have tested whether the mere inclusion of such judgments can alter recognition performance; results have been mixed. Hicks and Marsh (1999) found no difference in performance between standard recognition and a two-step old/new then RK test, but hit rate and FA rate were both increased when a one-step remember-know-new judgment was used, which they suggested was due to increased leniency when three response options have to be considered at once. Using a Deese–Roediger–McDermott false memory paradigm, Smith, Hunt, and Gallagher (2008) found that, compared with standard recognition, one-step remember/know/guess/new (RKGN) recognition led to no difference for true memories, but decreased false memories for items that had been visually presented. However, Smith et al. did not count guess responses as “old” when calculating hit and FA rates in the RKGN condition. Looking at their reported values, if they had included guesses, there would have been no difference in the FA rates with and without an RK judgment present, suggesting again that probing subjective experiences does not change recognition ability.

Naveh-Benjamin and Kilb (2012) asked younger and older adults to complete item (single word) and associative (paired words) recognition tests with and without RK judgments. They reported evidence that making RK judgments enhanced associative recognition performance for the older adults, but not for younger adults. However, Mulligan, Besken, and Peterson (2010) showed that inclusion of RK judgments can influence younger adults’ recognition. They found a modality match effect (superior recognition when items are shown in the same modality at study and test) only when an RK or source judgment was included at recognition, not on standard recognition. They concluded that RK instructions can focus participant’s attention toward perceptual aspects of stimuli, which can impact affect overall recognition rates when perceptual information is important. These mixed patterns of findings across studies suggest that whether the mere presence of RK judgments in a recognition task influences overall recognition performance depends on other factors, such as participants, stimuli, instructions, and definitions.^2^

Supporting previous work by Geraci et al. (2009), our findings suggest that the level of confidence inherent in definitions of know/familiar middle response option(s) influenced how participants used these responses. But our most important finding is that the change in the definition of the middle response option(s) also changed how remember and guess responses were used, even though definitions of remember and guess were kept constant. When the know definition included a sense of high confidence without recollection (RKG, RKFG, and RKfG conditions), fewer remember responses were made for deeply encoded items compared with when the middle response option was described as a familiarity-only-based subjective experience (RFG condition). There was also some evidence of this pattern for shallowly encoded items in the nonblocked test of Experiment 1, but there was no significant difference in how remember responses were used in the shallow block of Experiment 2. Use of the guess response was minimal (≤10% in all conditions, even for FAs), but changes to the definition of the middle option resulted in changes to use of the guess response, with patterns mirroring those for the remember response—fewer guess responses were made when the middle response(s) included a familiarity-only-based subjective experience (RKFG and RFG conditions).

The different test-list contexts in the two experiments also apparently resulted in differential responding across retrieval judgment conditions. In Experiment 1 there were significant changes to how remember, KF, and guess responses were used across retrieval judgment conditions for hits and FAs (though remember was not significant for FAs). In Experiment 2 on the recognition test for deeply encoded items there were no differences in proportion of FAs assigned to remember or KF, and on the recognition test for shallow-encoded items there were no differences in how either hits or FAs were assigned to remember. The removal of the deeply encoded items from the recognition test-list context for shallow-encoded items in Experiment 2 appears to have meant that although the different “middle option” definitions resulted in differences in how many shallow-encoded items participants assigned to the know and/or familiar and guess categories, this did not result in significant changes to proportion of items assigned to remember as it had done in Experiment 1, where the test-list context included both deep-encoded and shallow-encoded items. This pattern concurs with previous work suggesting that test-list context influences the functional definitions of remembering and knowing that participants use during a task (Bodner & Lindsay, 2003; Tousignant & Bodner, 2012; Tousignant et al., 2015).

That definitions and context influenced use of judgments fits with recent models of RK that suggest that both subjective experiences can be considered continuous (Ingram et al., 2012; Mickes, Seale-Carlisle, & Wixted, 2013; Wixted, 2007). Previous models had argued as to whether recollection—the process conceptualized as underlying remember judgments—should be thought of as high threshold (you either have it or you don’t; Yonelinas, 1994), or whether remember and know judgments simply reflected different criterion points on a continuum of memory strength or familiarity (e.g., Donaldson, 1996; Dunn, 2004, 2008; Wixted & Stretch, 2004). The continuous dual-process (CPD) model proposed by Wixted and Mickes (2010) suggests that recognition decisions are based on a continuous, unidimensional memory strength signal, but that this consists of information from separate continuous recollection and familiarity signals. Furthermore, it suggests that participants can query memory for the separable recollection and familiarity signals when asked to do so. Wixted and colleagues have provided support for this model in both recognition and recall paradigms through finding that remember and know judgments for which recognition accuracy and confidence had been equated were still differentiated by source memory accuracy (Ingram et al., 2012; Mickes et al., 2013). Their results suggest that the RK distinction is not simply one of memory strength (R being strong and K being weak), but that, when equal in strength, remember experiences reflect retrieval of contextual information, whereas know experiences reflect retrieval of item-only information based on a sense of context-free familiarity.

Our data patterns confirm the model’s suggestion that different definitions can affect where one sets their criteria for different subjective experiences (Ingram et al., 2012). In line with Mickes et al.’s (2013) suggestion that high-confidence and low-confidence know responses are based on different underlying processes (a recall-like process vs. perceptual fluency or automaticity), our results show that systematically varied confidence in know judgments influences how people use those judgments, and this affects where they put their cutoff for what determines a remember mnemonic signal and a know/familiar mnemonic signal. In accordance with previous research (Geraci et al., 2009; Ingram et al., 2012; Rotello et al., 2005), our findings suggest that how people approach an RK task is determined, in part, by the category distinctions researchers provide, and thus responses are not a pure reflection of underlying processes or retrieval of a particular form of information (Mickes et al., 2013).

The current experiments show that even small changes in how the middle nonrecollective response option is defined to participants can alter how participants use the remember response (and the guess response). The implications of these findings are that researchers should employ caution when interpreting others’ remember/know findings, particularly when evaluating patterns of responses across studies where RK instructions have not been provided in full.

Should we give up on the remember/know paradigm? No. Exploration of the subjective experiences associated with retrieval of different stimuli, under different instructions, or at differing levels of confidence/memory strength continues to be useful to increase understanding of how participants interpret mnemonic traces and how these influence recognition decisions (e.g., Uner & Roediger, 2018; Williams & Bodner, 2019). However, in line with recommendations put forward in a review of RK procedures by Migo et al. (2012), we suggest the best practice for researchers using the RK paradigm is to publish the exact instructions given to participants, which should include full definitions of the retrieval judgment options used in the experiment as well as describing how the researchers checked that participants understood and followed these instructions during the task. This will enable fully informed cross-experiment comparisons to be made.

## Footnotes

1 Order did not significantly interact with retrieval condition in any analysis (smallest *p* = .107 for analysis of hits made to shallow-encoded items in Experiment 1), so results for both experiments are reported collapsed across order.

2 It is also possible that the variations in findings were due to low statistical power.
